# Block Phenomena During Electric Micro-Stimulation of Pyramidal Cells and Retinal Ganglion Cells

**DOI:** 10.3389/fncel.2021.771600

**Published:** 2021-11-26

**Authors:** Sogand Sajedi, Andreas Fellner, Paul Werginz, Frank Rattay

**Affiliations:** Institute for Analysis and Scientific Computing, Vienna University of Technology, Vienna, Austria

**Keywords:** pyramidal cell, retinal ganglion cell, axon initial segment, neural stimulation, computer simulation, upper threshold, block, compartment model

## Abstract

Electric micro-stimulation of the nervous system is a means to restore various body functions. The stimulus amplitude necessary to generate action potentials, the lower threshold (LT), is well characterized for many neuronal populations. However, electric overstimulation above an upper threshold (UT) prevents action potential generation and therefore hinders optimal neuro-rehabilitation. Previous studies demonstrated the impact of the UT in micro-stimulation of retinal ganglion cells (RGCs). The observed phenomenon is mostly explained by (i) reversed sodium ion flow in the soma membrane, and (ii) anodal surround block that hinders spike conduction in strongly hyperpolarized regions of the axon at high stimulus intensities. However, up to now, no detailed study of the nature of these phenomena has been presented, particularly for different cell types. Here, we present computational analyses of LT and UT for layer 5 pyramidal cells (PCs) as well as alpha RGCs. Model neurons were stimulated in close vicinity to the cell body and LTs and UTs as well as the ratio UT/LT were compared. Aside from a simple point source electrode and monophasic stimuli also realistic electrode and pulse configurations were examined. The analysis showed: (i) in RGCs, the soma contributed to action potential initiation and block for small electrode distances, whereas in PCs the soma played no role in LTs or UTs. (ii) In both cell types, action potential always initiated within the axon initial segment at LT. (iii) In contrast to a complete block of spike conductance at UT that occurred in RGCs, an incomplete block of spiking appeared in PC axon collaterals. (iv) PC axon collateral arrangement influenced UTs but had small impact on LTs. (v) Population responses of RGCs change from circular regions of activation to ring-shaped patterns for increasing stimulus amplitude. A better understanding of the stimulation window that can reliably activate target neurons will benefit the future development of neuroprostheses.

## Introduction

Selective micro-stimulation of single neurons and, even more provoking, their subcellular structures such as the axon initial segment (AIS), soma or axon terminals are challenging for modern neuroprostheses. In the last decade, miniaturization of electronic components has enabled efficient stimulation of individual cells. For example, high-density micro-electrode arrays with a pixel pitch down to 25 μm have been developed for inner eye (retinal) prostheses (Mathieson et al., 2012). Penetrating electrodes might be even more successful for focal stimulation of the retina (Chen et al., 2020), the visual cortex, or other brain structures (Schmidt et al., 1996; Middlebrooks and Snyder, 2008; Nguyen et al., 2016).

In most cases of extracellular stimulation, cathodic stimulation requires less current amplitude to elicit a spike in a target cell than anodic stimulation (Ranck, 1975; Rattay, 1986, 1999). Therefore, cathodic pulses are often used for electric stimulation or, in order to avoid charge accumulation within the tissue, pseudo-monophasic cathodic pulses with a weak second anodic balancing phase are applied. However, for cathodic stimulation there is an intensity window with a lower threshold (LT) and an upper threshold (UT) for spike initiation and propagation. High-intensity cathodic stimulation of a nerve fiber causes strongly hyperpolarized regions on both sides of the electrode, blocking the propagation of an action potential (AP) generated in the central region close to the electrode. This phenomenon is called the anodal surround block or cathodic block (Katz and Miledi, 1965; Jankowska and Roberts, 1972; Roberts and Smith, 1973; Rattay and Aberham, 1993).

An intensity window for successful cathodic stimulation was also observed on cultured neurons before they developed neurites (Buitenweg et al., 2002). Moreover, stimulation of retinal ganglion cells (RGCs) with a micro-electrode close to the soma revealed an UT during high-amplitude stimulation with cathodic pulses (Boinagrov et al., 2012). A simplified computational model supported the hypothesis that the UT is caused by a reversal of sodium current flow in the soma when the transmembrane voltage exceeds the Nernst potential E_*Na*_ of sodium ions. Using a more detailed RGC model including dendrites, soma and axon, Rattay found cases in which the UT occurred in the soma while an AP was elicited in the axon which further propagated one-sided along the optic nerve (Rattay, 2014). This asymmetric firing is important for the interpretation of experiments, as the UT observation in the soma does not exclude spike conduction. The axonal activation during the somatic UT was also reported by Meng et al. (2018). In addition to the sodium reversal current, two crucial mechanisms were identified to cause the UT in the soma, namely the impact of strong potassium currents and the inactivation of sodium channels (Fellner et al., 2019).

In this modeling study, we used detailed three-dimensional geometries of two cell types representative for cortical and retinal stimulation: rat pyramidal cells (PCs) from the somatosensory cortex and mouse alpha RGCs. Our analysis aimed to identify the somatic contribution to AP generation at LT as well as blockage of spikes at UT during micro-stimulation close to the cell body. We examined AP initiation with a focus on the AIS and spike propagation in light of complex axon collateral structures. Finally, we studied realistic electrode and pulse properties as well as the impact of electrode positioning.

## Materials and Methods

### Model Neurons

**FIGURE 1:**
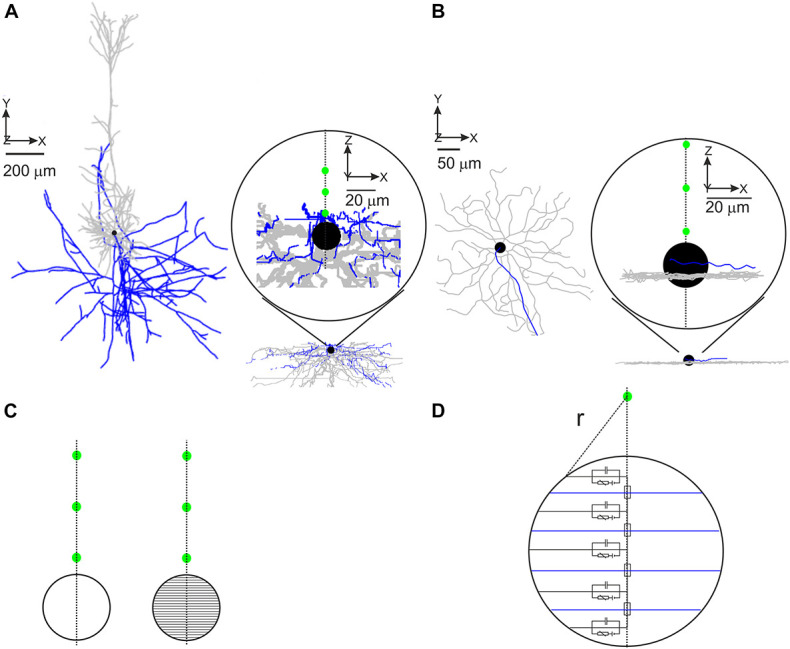
Implementation of realistic model neurons. Representative PC **(A)** and RGC **(B)** model geometries. Only the first three investigated electrode positions (15, 30, and 45 μm) are shown as green circles in the insets. Dendrites shown in gray, axon in blue, and soma in black. **(C)** The soma was modeled as a single compartment (left) or was split into 41 compartments (right). The main axis of the multi-compartment soma was oriented toward the electrode. The green circles represent the first three investigated electrode positions. **(D)** Scheme of the segmented soma (only five compartments for better visibility) with the Euclidian distance (*r*) to the electrode (green) used to calculate the extracellular potential.

Eight reconstructed morphologies of layer 5 pyramidal neurons of rat somatosensory cortex were downloaded from an online database^1^ (Ascoli, 2006; Hay et al., 2013; Cohen et al., 2020); a model cell is shown in Figure 1A. Soma diameter was set to 20 μm in all model PCs. Axonal trees were split into hillock, AIS, unmyelinated axon, nodes of Ranvier, and myelinated axon (see Table 1). The hillock (*L* = 0–2 μm) was attached to the soma followed by the AIS (*L* = 35–48 μm) and an unmyelinated axon section up to the first axonal bifurcation (100–150 μm from the soma). Following axonal branches were split into nodes of Ranvier (0.5–1 μm) and internodes (*L* = 100 × section diameter; Rushton, 1951). Nodes of Ranvier were placed at the beginning of each branch as well as at the end of myelin sections except at terminal branches. The length of each node of Ranvier was dependent on the length of the adjacent myelin section; this was done to prevent self-spiking that could result from a too large nodal area.

**TABLE 1:** Ionic current densities along the neural membrane for PC models based on data from Mainen and Sejnowski (1996) and Almog and Korngreen (2014).

	**Unit**	**SOMA**	**DEND**	**AXON**				
				**Hill**	**AIS**	**Unmy**	**Node**	**Inter**
g_*Na*_	pS/μm^2^	352	56	6000	30000	1000	30000	352
g_*K,fast*_	pS/μm^2^	332	28	1000	1000	332	1000	332
g_*K,slow*_	pS/μm^2^	206	3.79	1500	1500	206	1500	206
g_*H*_	pS/μm^2^	2.51	118					
g_*bK*_	pS/μm^2^	0.64	1.23					
g_*sK*_	pS/μm^2^	3.18	0.52					
p_*HVA*_	μm/s	0.93	1.56					
p_*MVA*_	μm/s	31.5	4.9					

*g_*Na*_, g_*K,fast*_, g_*K,slow*_, g_*H*_, g_*bK*_, and g_*sK*_ represent the maximum conductivities of sodium, fast inactivating potassium, slow inactivating potassium, hyperpolarization-activated cation, small-conductance Ca^2+^-gated potassium, and large-conductance Ca^2+^-gated potassium channels, respectively. p_*HVA*_ and p_*MVA*_ are the permeabilities of the Ca^2+^ high- and medium voltage-gated channels.*

*The dendritic conductivities decreased with increasing distance from the soma, for more details see Almog and Korngreen (2014).*

34 reconstructed morphologies of mouse alpha RGCs were taken from a previous study (Werginz et al., 2020); Figure 1B shows a representative model cell. Each model neuron was divided into dendrites, soma (*D* = 14–24 μm), hillock (*L* = 10–47 μm), AIS (*L* = 12–33 μm), and distal (unmyelinated) axon (*L* ∼ 1000 μm).

For each cell, the soma was either modeled as a single spherical compartment or as a sphere approximated by 41 cone-shaped compartments. For the multi-compartment soma approach (Figure 1C), the main axis of the soma was always pointing toward the stimulating electrode to study the gradient of the applied electric field across the soma (Fellner et al., 2019). Responses to intracellular and extracellular stimulation in both cell types were performed in NEURON 7.8 (Carnevale and Hines, 2006); Python 3.8^2^ was used to control the simulations. Compartment length was between 1 and 2 μm in the axon and below 10 μm in dendritic sections. Extracellular stimulation was modeled *via* NEURON’s “extracellular” mechanism. A monophasic cathodic pulse with a duration of 0.1 ms was used in the majority of all experiments. Charge-balanced biphasic pulses with different cathodic/anodic ratios were used in a subset of simulations.

### Biophysical Properties

For PCs, the biophysical properties (i.e., ion channel kinetics and densities, Table 1) of the soma and dendrites were specified based on experimental data (see Table 1 and cell 5 data of Almog and Korngreen, 2014) and the axon kinetics were adapted from Mainen and Sejnowski (1996). Leak conductivity, intracellular resistivity, and specific membrane capacitance were set to 0.39 pS/μm^2^, 120 Ω.cm, and 0.6 μF/cm^2^, respectively, except for the myelinated part of the axon. The nodes of Ranvier had an increased leak conductivity of 200 pS/μm^2^, whereas the myelinated internode had a lowered membrane capacitance of 0.04 μF/cm^2^. Model temperature was set to 34°C. Model baseline function, including action potential initiation and (back)propagation as well as spike shape, was tested by intracellular current injections into the PC soma and compared to Almog and Korngreen (2014).

Biophysical properties of RGCs were slightly modified from previous studies (Fohlmeister et al., 2010; Werginz et al., 2020; Table 2) without changing baseline function, i.e., AP initiation in the AIS, forward and backward propagation of spikes into the distal axon and somatodendritic compartment, respectively. Leak conductivity, intracellular resistivity, and specific membrane capacitance were set to 2.5 pS/μm^2^, 143 Ω.cm, and 1 μF/cm^2^, respectively. Model temperature was set to 33°C.

**TABLE 2:** Ionic current densities along the neural membrane for RGC models based on data from Fohlmeister et al. (2010) and Werginz et al. (2020).

	**Unit**	**SOMA**	**DEND**	**AXON**		
				**Hill**	**AIS**	**Unmy**
g_*Na1.2*_	pS/μm^2^	650	650	1625	0	1000
g_*Na1.6*_	pS/μm^2^	0	0	0	1625	0
g_*K1.2*_	pS/μm^2^	350	350	625	0	700
g_*K1.6*_	pS/μm^2^	0	0	0	625	0
g_*Ca*_	pS/μm^2^	15	15	15	15	15
g_*K,Ca*_	pS/μm^2^	1.5	1.5	1.5	1.5	1.5

*g_*Na1.2*_, g_*Na1.6*_, g_*K1.2*_, g_*K1.6*_, g_*Ca*_, and g_*K,Ca*_ represent the maximum conductivities of sodium Na_*v*_1.2/1.6, their corresponding potassium, calcium, and Ca^2+^-activated potassium channels, respectively.*

### Extracellular Potentials

Stimulating electrodes were placed in close vicinity of the soma within distances of 15, 30, 45, 60, 100, and 200 μm to the soma center (Figures 1A,B, green circles). The extracellular potential *V*_*e*_ was calculated for a point source in a homogeneous infinite medium. In addition, disk electrode stimulation was applied to RGCs in a semi-infinite medium. The extracellular resistivity *ρ_*e*_* was smaller for cortical tissue (300 Ω.cm; Rattay and Wenger, 2010) than for retinal tissue (1000 Ω.cm; Werginz and Rattay, 2016).

For point source stimulation the extracellular potential was calculated as (Rattay, 1999)


$$V_{e}=\frac{\rho_{e}\,I_{s\,t\,i\,m}}{4\,\pi \,r}$$

with *I*_*Stim*_ being the stimulus current applied to the electrode and *r* representing the Euclidian distance from the compartment center to the electrode. In the multi-compartment soma configuration, *r* was computed as the distance to the compartment surface (Figure 1D).

The electric potential generated by a disk electrode was calculated as (Newman, 1966; Wiley and Webster, 1982; Rattay, 1988)


$$V_{e}=\frac{\rho_{e}\,I_{s\,t\,i\,m}}{2\,\pi \,a}\,\arcsin\,\left(\frac{2\,a}{\sqrt{{\left(r-a\right)}^{2}+z^{2}}+\sqrt{{\left(r+a\right)}^{2}+z^{2}}}\right)$$

with *a*, *r*, and *z* being the electrode radius, the radial, and the axial distance to the electrode, respectively.

### Statistical Analysis

Comparison between two groups were performed by the Kruskal-Wallis test. Significance levels were set as follows: *p* < 0.05 ^∗^, *p* < 0.01 ^∗∗^, *p* < 0.001 ^∗∗∗^. Boxplots use standard notation (1st Quartile, Median, 3rd Quartile). All statistical analyses were performed in Python 3.8.

## Results

### Somatic Contribution to Lower and Upper Thresholds

**FIGURE 2:**
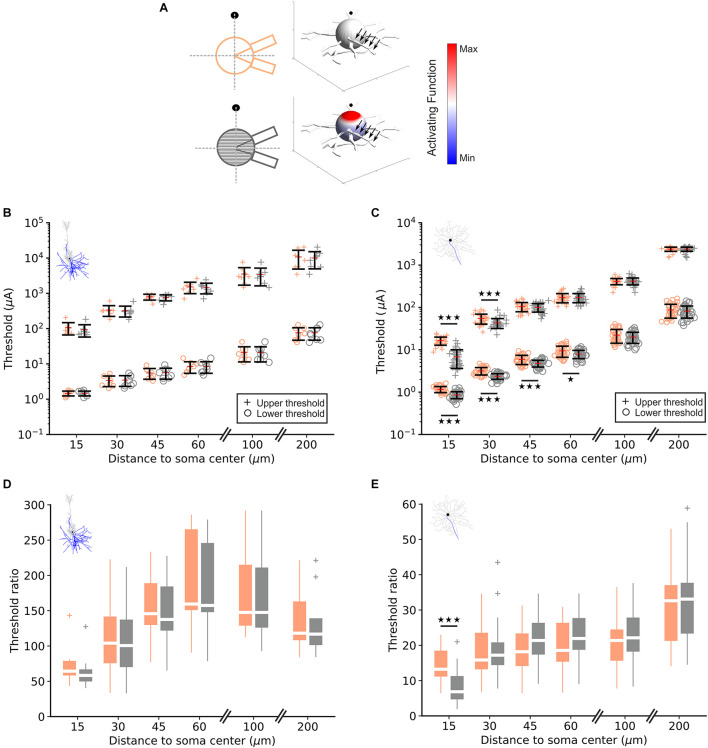
Lower and upper threshold in RGC and PC models. **(A)** For one RGC and both soma configurations (left), each compartment is color-coded by its activating function in response to a 1 μA cathodic pulse (right). The point source electrode is shown as filled black circle/sphere, black arrows indicate the axon. **(B)** Computed lower (“o”) and upper (“+”) thresholds for the single- (orange) and multi-compartment soma (gray) in eight PCs for different electrode distances. **(C)** Same as panel **(B)** for 34 RGCs. **(D)** Threshold ratio UT/LT for PCs at different electrode distances. **(E)** Same as panel **(D)** for RGCs; note the different y-scales in panels **(D,E)**.

To examine the contribution of the soma to LT and UT, model neurons were either equipped with a soma consisting of a single sphere or of 41 truncated cone compartments (Figures 1C, 2A left). The right part of Figure 2A shows the activating function, a predictor for excitation along the neural membrane (Rattay, 1999), for a cathodic electrode current of 1 μA. For the multi-compartment soma, the activating function is positive (red, indicating depolarization) in compartments close to the electrode, whereas compartments further away from the electrode have a negative activating function (blue) indicating hyperpolarization. In contrast, for the single-compartment soma, the activating function is small (white), resulting from no reflection of the extracellular potential gradient across the soma.

LTs and UTs in PCs (*n* = 8) and RGCs (*n* = 34) were computed at point source distances of 15, 30, 45, 60, 100, and 200 μm to the soma center (Figures 2B,C). As expected LTs and UTs increased with electrode distance; LTs were in similar ranges in RGCs and PCs for small distances (∼1 μA for 15 μm distance). However, in PCs LTs were higher but still within an order of magnitude compared to RGCs for larger distances. In contrast, UTs were almost 10-fold larger in PCs vs. RGCs.

No significant difference between both soma configurations, neither in LTs nor in UTs, was observed in PCs for all distances indicating little contribution of the *trans-*somatic electric field to UTs (Figure 2B, orange vs. gray). In contrast, for RGCs, LTs in the multi-compartment configuration were significantly lower than in the single-compartment soma model for electrode distances ≤60 μm. Also, UTs were significantly lower in the multi-compartment soma configuration for electrode distances of 15 and 30 μm (Figure 2C, orange vs. gray).

Threshold ratios (UT/LT) in PCs were calculated for the single- and multi-compartment soma configuration (Figure 2D). The ratios were smallest for the shortest investigated electrode distance, increased for distances up to 60 μm and decreased again for electrode-to-soma distances of 100 and 200 μm. No significant difference was found in ratios between both soma configurations at any examined electrode distance. Figure 2E displays threshold ratios for RGCs for single and multi-compartment soma configurations, respectively. Threshold ratios increased monotonically with electrode distance and a significant difference between both soma configurations could be observed in RGCs for the closest electrode distance suggesting a significant contribution of the soma in determining the stimulus window.

We were also interested in whether single morphological features influence LTs, UTs as well as threshold ratios for a given distance. We computed linear correlations between single anatomical parameters (e.g., AIS length, soma diameter, etc.); *r*^2^ values for each correlation are shown in Supplementary Tables 1,2. We did not observe a clear trend for any feature tested, with an exception of PC dendritic area vs. LT that resulted in *r*^2^ values >0.4 for all distances (Supplementary Table 1). Additionally, AIS length was somewhat correlated to thresholds and threshold ratios for small electrode distances, similar to results obtained by previous studies that showed a correlation between AIS length and LTs (Jeng et al., 2011; Werginz et al., 2020). These results indicate that LTs, UTs as well as threshold ratios are not dependent on single anatomical properties but to a combination of anatomy, biophysics as well as axonal geometry relative to the electrode location.

In summary, we found up to 300-times higher UTs in PCs compared to their LTs whereas threshold ratios were below 50 for all electrode distances in RGCs. A significant soma contribution was observed in both LTs and UTs for RGCs, particularly for small electrode distances, in contrast to a negligible soma contribution in PCs regardless of electrode distance.

### Partial Upper Threshold in Pyramidal Cells

**FIGURE 3:**
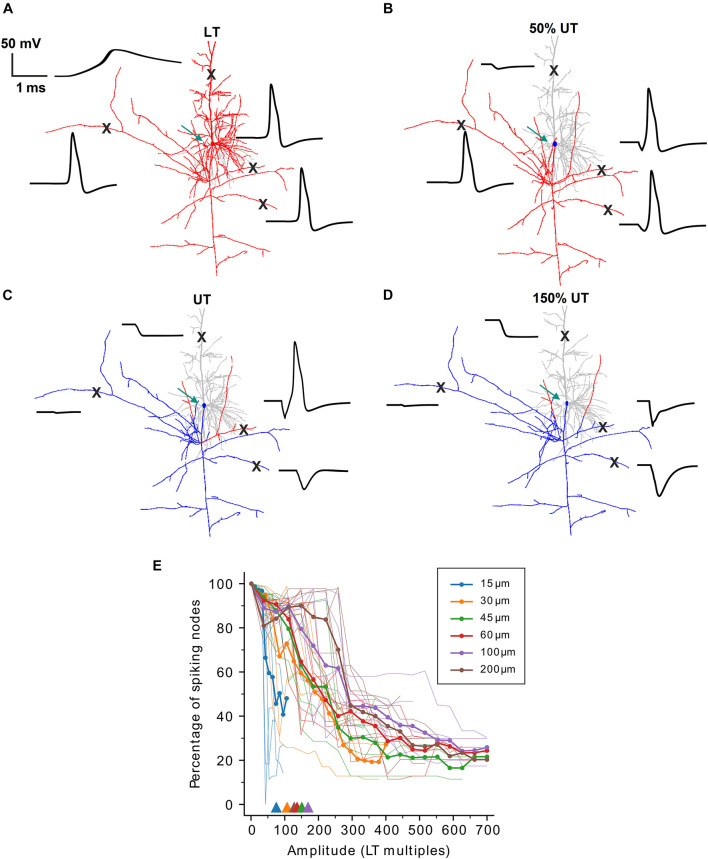
Partial spiking activity during high-amplitude stimulation in PCs. **(A)** Neuron shape plot showing portions along the neural membrane which generated an AP (red) and that were not excited (gray) when stimulated at LT. Membrane voltage over time (black) is shown for four locations (“X”) along the PC. **(B–D)** Similar as panel **(A)** but for amplitudes of 50% UT **(B)**, UT **(C)**, and 150% UT **(D)**, respectively. Regions with an AP are shown in red, regions without an AP in gray (dendrites) or blue (axon and soma). Green arrows indicate the electrode location 15 μm distant to the soma. **(E)** The percentage of spiking nodes of Ranvier is plotted against stimulus amplitude (in multiples of LT) for all six investigated electrode distances. Thin lines indicate the spiking node percentage for single cells, thick lines indicate population means. Triangles show the mean thresholds ratios from Figure 2D for different electrode distances.

In our initial experiments, spiking activity was detected in the distal axon in RGCs as it is the sole output pathway in these cells. PCs, however, have complex axons with numerous collaterals, and we decided to detect APs at the first node of Ranvier approximately 100–150 μm distant to the soma where the axon starts to bifurcate. In a second set of simulations, we questioned whether partial spiking, i.e., APs in one part of the axon but no spiking in other portions of the cell, could occur in PCs. Surprisingly, a complete block did not occur in any of the eight investigated PCs, but depending on the electrode distance to the soma, at least small portions of the cell generated an AP, even at amplitudes above previously determined UTs. Figure 3 demonstrates this partial block phenomenon for one model cell (PC7) when the electrode was at a distance of 15 μm. At LT, the axon and soma generated an AP which actively backpropagated into the dendritic tree [Figure 3A, note the broad dendritic calcium spike (Almog and Korngreen, 2014)]. When the pulse amplitude was set to 50% of the UT the axonally initiated AP did not propagate across the soma and backpropagation into the dendrites failed (Figure 3B). At stimulus levels equal to and higher than UT, some parts along the axon still generated APs while most portions of the cell were in a blocking condition (Figures 3C,D).

To quantify the partial spiking in axon collaterals, we computed the percentage of spiking nodes of Ranvier at stimulus amplitudes above LT (Figure 3E). For the smallest electrode distance of 15 μm, similar to the results from Figure 2D (see Figure 3E, triangles), the percentage of spiking nodes dropped rapidly when stimulus amplitude exceeded ∼50 × LT. For larger distances the spiking percentage decreased but eventually plateaued at approximately 25% indicating partial spiking even at stimulus amplitudes >500 × LT.

Taken together, no complete block was observed in PCs for all examined distances and AP blockage was mostly observed in regions with strong hyperpolarization in response to high-amplitude stimulation. The partial block is mostly specific to extracellular stimulation of the cell and originates from an inhomogeneous reflection of electric field on the transmembrane voltage of axon collaterals and inhomogeneous activating function.

### Axon Initial Segment-Induced Spiking During Micro-Stimulation

**FIGURE 4:**
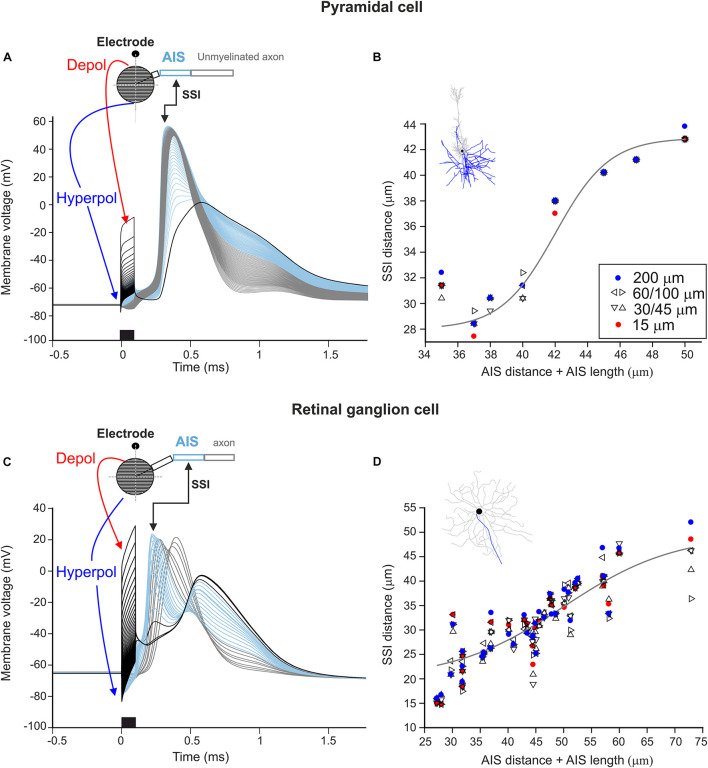
Site of spike initiation in AIS for point source stimulation. **(A)** Membrane voltage of 41 soma compartments (black lines), AIS compartments (blue), other axon compartments (gray) as a function of time. The black kinked arrow indicates the site of spike initiation (SSI). The red and blue arrows show the location of soma compartments which are de- and hyperpolarized most strongly. The stimulus (cathodic, 0.1 ms) is shown at the bottom, the point source electrode was located 15 μm above the soma. **(B)** For all electrode distances of the eight PCs, the distance between the soma and the site of spike initiation is plotted versus the distance between the soma and the distal end of the AIS (i.e., AIS distance + AIS length). The gray curve indicates the best-fit logistic function (*r*^2^ = 0.91). **(C)** Same as panel **(A)** for a representative RGC model neuron. **(D)** Same as panel **(B)** for 34 RGCs, the gray curve indicates the best-fit logistic function (*r*^2^ = 0.74).

In the next set of experiments, we were interested in the location where APs are initiated during stimulation close to the soma. The site of spike initiation (SSI) was defined as the compartment whose membrane voltage first crossed 0 mV. The SSI was examined in the multi-compartment soma configuration. When applying a monophasic cathodic pulse close to the soma (15 μm distance), following the activating function (cf. Figure 2A), a distinct polarization pattern of the somatic compartments could be observed. For both cell types, somatic compartments close to the stimulating electrode were strongly depolarized during the pulse whereas compartments on the far side of the soma were hyperpolarized (Figures 4A,C, red and blue arrows). Interestingly, the strong depolarization at the soma did not result in an AP directly but compartments along the proximal axon initiated the spike (Figures 4A,C, light blue). This region is called the axon initial segment and, due to its high density of sodium channels, has been shown to be the SSI during a variety of stimulation conditions in both PCs and RGCs (Rattay and Wenger, 2010; Werginz et al., 2014). The AIS-induced AP subsequently propagated bidirectionally into the distal axon as well as backward into the soma and dendrites (Figures 4A,C, thick black line).

The experiment was repeated for all model neurons at electrode distances of 15, 30, 45, 60, 100, and 200 μm. The SSI was confined to the AIS in all cases, with small variations for different electrode distances. This is shown by plotting the distance between the SSI and the soma vs. the sum of the distance between the AIS and the soma and the length of the AIS (Figures 4B,D). The solid gray lines show the best-fit logistic functions with *r*^2^ values of 0.74 and 0.91 for RGCs and PCs, respectively.

Our results show that APs are always initiated within the AIS for micro-stimulation in the vicinity of the soma, regardless of electrode-to-soma distance. For short AIS lengths, the AP initiated close to the distal end of the AIS, whereas in cells with longer AIS which are located far from the soma the SSI was shifted to the center of the AIS.

### Stimulus Parameters Affect Threshold Ratios

**FIGURE 5:**
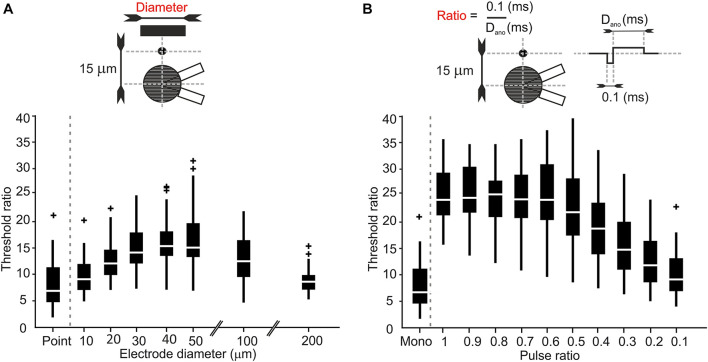
Electrode size and pulse configuration affect threshold ratios in RGCs. **(A)** Threshold ratio vs. electrode diameters ranging from 10 to 200 μm. **(B)** Threshold ratio of monophasic and charge balanced biphasic pulses plotted vs. the ratio 0.1/D_*ano*_.

So far, our simulations were restricted to stimulation with a point source and monophasic pulses from defined electrode locations. However, parameters such as electrode size and pulse duration cannot be chosen freely in neural implants due to hardware, software, and energy constraints. The placement of stimulating electrodes is also limited by constraints of the surrounding biological tissue. In the retina, for example, implants can be placed on the epiretinal surface to stimulate RGCs directly. In such implants an array of disk electrodes interfaces with the targeted RGCs. Therefore, the impact of the size of disk electrodes on LTs, UTs and the UT/LT ratios was examined. The electrode-to-soma distance was set to 15 μm as in our previous simulations as the strongest effect of the *trans-*somatic electric field on LTs and UTs was observed at smallest distances. Figure 5A shows UT/LT ratios for electrode diameters ranging from 10 to 200 μm and compares them to the point source electrode results. For electrode diameters in the range of 10–50 μm threshold ratios increased, however, for the largest electrode tested we found decreasing threshold ratios that were in a similar range as for point source stimulation.

To prevent potentially tissue damage due to charge accumulation, most studies use charge-balanced biphasic pulses. Therefore, we studied threshold ratios when a biphasic cathodic-first pulse was applied. The cathodic phase duration was held constant at 0.1 ms, whereas the following anodic pulse duration was varied from 0.1 up to 1 ms with its amplitude adjusted for charge-neutral stimulation. Threshold ratios are shown for phase duration ratios (D_*cat*_/D_*ano*_) ranging from 0.1 to 1 and compared with the monophasic pulse used in our previous simulations (Figure 5B). Our results indicate up to threefold higher threshold ratios for symmetric and close to symmetric pulse shapes (pulse ratio > 0.6), whereas this effect decreased for longer charge balancing pulses. For the longest anodic pulse of 1 ms the biphasic stimulus became pseudo-monophasic and its UT/LT ratio was close to the monophasic case.

**FIGURE 6:**
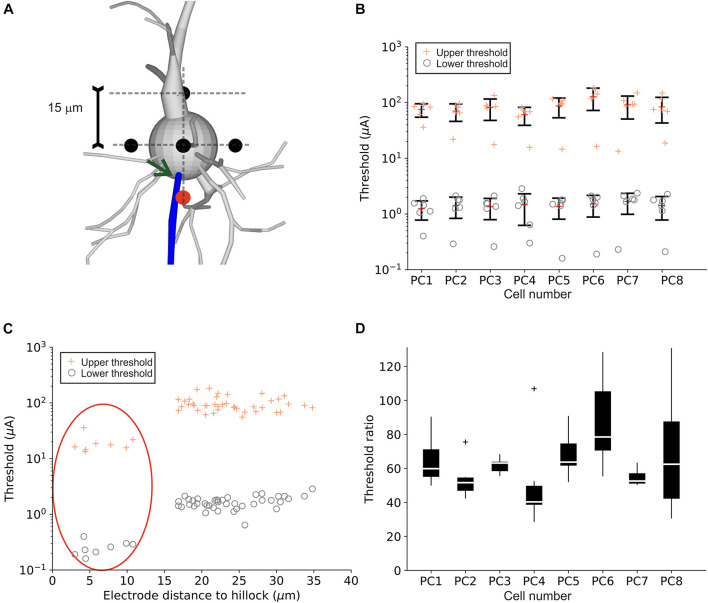
Upper and lower threshold for different point source locations close to the PC soma. **(A)** Six electrode positions (black and red circles) in a distance of 15 μm to the soma center were investigated for the eight PC model neurons. The red circle indicates the electrode positions closest to the hillock (indicated by arrow). **(B)** LTs (“o”) and UTs (“+”) are shown for individual cells. **(C)** LTs (“o”) and UTs (“+”) plotted as a function of electrode distance to the hillock. Points within the red ellipse correspond to the red electrode location in panel **(A)**. **(D)** Threshold ratios for all eight PCs for all electrode locations.

Targeted stimulation of PCs with neural implants is challenging as the precise electrode location cannot be determined during surgery. The inserted micro-electrodes are placed within the targeted cortical layer(s) and thus can be located randomly in space relative to PC somata. In order to quantify the effect of the electrode-to-soma positioning, LTs, UTs as well as threshold ratios were calculated in the eight PC model neurons for six electrode locations around the soma. Electrode distance was set to 15 μm to the center of the multi-compartment soma (Figure 6A). LTs and UTs for all investigated positions are shown for each cell in Figure 6B. Interestingly, for each of the eight model neurons tested, one electrode location resulted in significantly lower LTs and UTs (outliers in Figure 6B). More detailed analysis revealed that these outliers were linked to the electrode distance to the hillock (Figure 6C). Similar LTs and UTs were observed for all positions, except for the closest electrode location to the axon hillock, which resulted in the lowest LTs and UTs (red ellipse). Figure 6D shows threshold ratios of the six investigated electrode locations for each cell. Median ratios ranged from 40 to 80 similar to threshold ratios for an electrode distance of 15 μm (cf. Figure 2D).

### Population Response of Retinal Ganglion Cells to Supra-Threshold Stimulation

**FIGURE 7:**
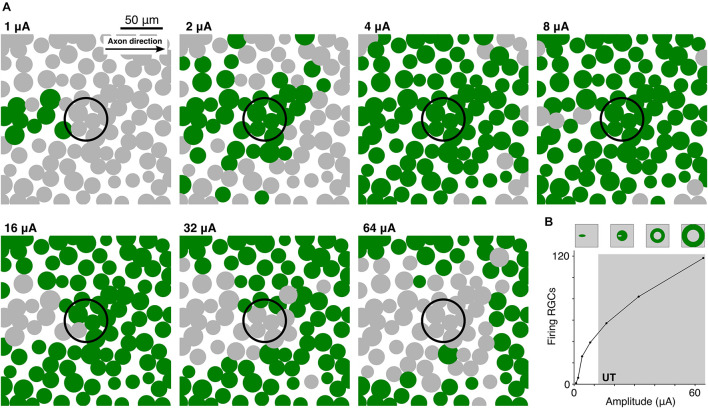
High-amplitude stimulation results in distorted RGC activation patterns. **(A)** Top view of 100 RGC somas (filled circles) within a 200 × 200 μm patch of the retina. Each RGC was rotated so that its axon pointed toward the right. RGCs were stimulated by a 50 μm disk electrode (black unfilled circle) located 15 μm from the epiretinal surface at amplitudes ranging from 1 to 64 μA. For a given amplitude, green circles indicate cells which fired an action potential whereas gray RGCs did not respond. **(B)** 200 RGCs were stimulated within a region of interest 1 × 1 mm in size. The number of RGCs that fired an action potential is plotted versus the stimulus amplitude. Schematics on top indicate the transition of the activation pattern with increasing amplitude.

In a final set of experiments, we explored how the UT affects the activation pattern of a population of RGCs. Figure 7A shows the transition from LT to UT for 100 RGCs distributed on the epiretinal surface when stimulated by a 50 μm disk electrode. The pulse was biphasic with a pulse ratio of 0.1, i.e., a 0.1 ms cathodic stimulus followed by a 1 ms balancing pulse. Small amplitudes led to focal activation of RGCs which were located slightly offset from the stimulating electrode (Figure 7A, 1 and 2 μA). This can be explained by the low-threshold region at the distal AIS in RGCs (Fried et al., 2009; Jeng et al., 2011; Werginz et al., 2020). Increasing the stimulus amplitude led to an enlarged region of activation (Figure 7A, 4 and 8 μA). At high amplitudes the UT resulted in a different activation pattern with RGCs that had lowest thresholds being prevented from firing APs first (Figure 7A, 1 vs. 16 μA). Highest amplitudes generated a ring of activated RGCs around the stimulating electrode creating a non-responding region around the stimulating electrode (Figure 7, 32 and 64 μA). We were also interested in the total number of activated RGCs for different stimulus amplitudes. Figure 7B shows a monotonic increase of activated cells when the stimulus amplitude was increased from 1 to 64 μA. Interestingly, the number of activated cells still increased when RGCs close to the electrode were already blocked (gray shading). However, despite a larger number of activated cells at higher amplitudes, the different activation pattern (circular versus ring-shaped, Figure 7B, top) is not expected to generate well-defined visual percepts.

## Discussion

During extracellular electric stimulation axons are the most sensitive elements of a neuron for spike initiation (Porter, 1963; Nowak and Bullier, 1998; Rattay and Wenger, 2010). Typically, axon stimulation requires about five times lower intensities for cathodic versus anodic pulses (BeMent and Ranck, 1969; Rattay, 1990). Consequently, to save energy, cathodic stimulation is often preferred in neuroprosthetics, e.g., as pseudo-monophasic pulses in deep brain and spinal cord stimulation. The existence of an upper limit/threshold (UT) for cathodic axon stimulation was detected and quantified by a ratio UT/LT of about 3 (Katz and Miledi, 1965) and a ratio of 8–10 during micro-stimulation of myelinated fibers in the spinal cord (Roberts and Smith, 1973; Ranck, 1975).

Spike initiation in dendrites is demanding because of their low sodium channel density (Gasparini et al., 2004; Rattay and Wenger, 2010; Rattay et al., 2012). An increased sodium channel density in the soma (Table 2) makes it a potential candidate (i) to play a role in spike initiation as the depolarized region of the soma activates the low threshold sodium channels in the AIS, and (ii) for influencing the UT because of the strong gradient of the electric field across the soma during micro-stimulation. A large gradient of the applied electric field is needed to depolarize portions of the somatic membrane and the primarily depolarized region is smaller than half of the somatic surface (Figure 2A; Fellner et al., 2019). Such large gradients are expected in the vicinity of micro-electrodes.

As shown by experiments, a micro-electrode in a distance of about 25 μm from an RGC soma resulted in a LT of 3 μA and an UT at 18 μA, i.e., an UT/LT ratio of 6, if both LT and UT are defined by the rule 50% of the pulses elicit spikes, whereas the ratio is about 10 for stronger limits, e.g., rare spiking similar to spontaneous firing at LT and UT (Boinagrov et al., 2012). We obtained comparable UT/LT ratios in the investigated model RGCs which increased with electrode distance (Figure 2E). In order to see the contribution of the soma to spike generation, the simulations were executed with a single- and a multi-compartment model of the soma. The difference in the statistical plots of both evaluations is an indicator that the soma contributes to spike generation and suppression. This difference was significant in RGCs for LTs and UTs when electrode distance was smaller than 60 and 30 μm, respectively (Figure 2D). In contrast, no significant difference was found in PCs between both soma configurations, neither in LTs nor in UTs (Figure 2B).

As no experimental UT studies are available for PCs, we speculate that the small impact of the PC soma to LTs and UTs is based on anatomical differences in comparison to RGCs, these are (i) a larger number of dendrites has to be maintained by the PC soma with intracellular current flow during somatic excitation and (ii) the PC axon has many branches in various directions (Figure 1). The complex cell geometry of PCs hinders the complete block in all axon branches (Figure 3) and reduces the UT/LT ratio slightly if certain axon branches are removed (not shown).

Some of our observations are in line with previous results showing that during extracellular stimulation of cortical neurons axonal firing could be observed while the soma, dendrites, AIS, as well as the first node of Ranvier were blocked artificially (Nowak and Bullier, 1996). For stimulation of PCs around the soma (up to 200 μm away in our case) we observed that partial UTs are related to the arrangement of axonal branches and their distance to the stimulating electrode, which affected UTs, whereas it had little impact on LTs. In RGCs, on the other hand, a complete UT always arises from either a somatic UT for close electrode distances with smaller threshold ratios (up to 15) or an anodal surround UT of the axon for larger electrode distances with higher threshold ratios (up to 60).

We show that at LT, APs always initiated within the AIS in both cell types as in agreement with various studies using intracellular stimulation or synaptic excitation (Palmer and Stuart, 2006; Shu et al., 2007; Yu et al., 2008; Bender and Trussell, 2012). This is also in line with previous studies showing the AIS is the most sensitive region for electric stimulation (Fried et al., 2009; Jeng et al., 2011; Werginz et al., 2020); however, in our study, stimulation was always applied close to the soma and not directly above the AIS. The somatic polarization leads to axial currents depolarizing the AIS, which subsequently initiate the AP because of its high density of sodium channels. In contrast to an experimental study that shows the distal end of the AIS (∼35 μm) to be the site of AP initiation in layer 5 PCs (Palmer and Stuart, 2006), we demonstrate that with increasing the AIS length, the site of AP initiation was shifted toward the center of AIS in both cell types (Figure 4).

For RGCs, we also tested realistic electrode geometries as well as pulse parameters and how these affect LTs and UTs. Our findings suggest that small micro-electrodes up to 50 μm in diameter increase the stimulation window as UTs become larger. On the other hand, the largest electrode diameter tested (200 μm) resulted in low threshold ratios. In retinal implants, the optimal electrode size is still under debate; however, our results indicate that UTs are not likely to play a significant role during stimulation, independent of electrode size. Similarly, symmetric charge-balanced pulses, as mostly applied in current retinal implants, increase the threshold ratio up to 25. Pseudo-monophasic pulses, on the other hand, resulted in (low) threshold ratios similar to monophasic pulses. In PCs, LTs and UTs but not threshold ratios were highly dependent on the relative arrangement between electrode and target neuron with electrode locations close to the axon hillock leading to both lowest LTs and UTs (Figures 6B,C). In cortex, the large number of PCs surrounding a stimulating electrode can therefore have different LTs and UTs but similar threshold ratios.

For actual applications of electric stimulation in neuroprosthetics, the question arises which stimulation configuration maximizes the stimulation window, i.e., the amplitude range which can elicit an AP. In RGCs, our results suggest that stimulation from a large distance (200 μm) with an intermediate electrode size (50 μm) and biphasic symmetric charge balanced pulses (pulse ratio = 1) will maximize the stimulation window. However, stimulation from a relatively large distance from the epiretinal surface will not achieve high spatial resolution and therefore limit clinical outcome. For PCs, on the other hand, stimulation from intermediate distances (60 μm) resulted in highest threshold ratios, however, our results show a strong dependency of thresholds and threshold ratios from the axonal geometry in PCs. Therefore, we cannot make a general statement for PCs which stimulus configuration maximizes threshold ratios.

The models used in this study include assumptions that simplify the underlying mechanisms. Although our presented models are detailed descriptions of the anatomy and biophysics of RGCs and PCs, there are still several shortcomings that should be mentioned. In all our computations, the extracellular medium, i.e., retinal or cortical tissue, was assumed to be homogeneous. In reality, however, neural tissue was shown to be non-homogeneous which will lead to distorted electric field within the tissue. For small electrode-to-cell distances, we assume that the non-homogeneity will not have a strong influence on our results, however, we cannot rule out that for large distances this effect will alter our results moderately. The majority of the results shown in this study were computed with a simplified point source approach which does not accurately mimic the clinical situation for retinal and cortical stimulation. However, electric fields generated by a point source become similar to fields generated by a disk electrode for cases when the distance between target neuron and the electrode is larger than the electrode diameter (see Werginz et al., 2020). Therefore, in the retina the point source approach is comparable to stimulation with small disk electrodes [<20 μm, (Grosberg et al., 2017)] even when the electrode-to-cell distance is small. The retina as well as the brain consist of a large number of cell types which a single study cannot investigate. We chose two major cell types of the retina and cortex, however, cannot rule out that other cell types will respond differently to high-amplitude stimulation and therefore will have different threshold ratios than we report here.

Overall, our results indicate that an upper threshold exists during electric micro-stimulation in PCs and RGCs. The practical implications of an upper threshold are still under debate, especially because of the lack of experimental and clinical data. Here, we show that in RGCs UTs are at least as high as five times LTs for smallest electrode distances (Figure 1). Our simulations of population responses with realistic electrodes and pulse configurations resulted in UTs of approximately 15 × LT (Figure 7). Based on these results it is unlikely that UTs will have strong practical implications in retinal implants. However, in this study we only investigated one class of cells (alpha RGCs); whether other classes of cells will have different responses to high-amplitude stimulation and/or lower UTs is unknown and therefore requires additional experimental and computational studies. PCs were shown to have higher threshold ratios than RGCs in the range of 50–200 (Figure 1). Therefore, our results suggest that UTs will not have practical implications in stimulation of cortical tissue. However, we show that the arrangement of axon collaterals is prone to generate partial firing of APs (Figure 3) which makes it more difficult to derive general statements about the outcome of high-amplitude stimulation in the cortex based on computational analyses.

## Data Availability Statement

The original contributions presented in the study are included in the article/Supplementary Material, further inquiries can be directed to the corresponding author/s.

## Author Contributions

SS contributed to the conception and design of the study, implementation of computational models as well as data analysis, visualization, and writing of the manuscript. AF contributed to the implementation of computational models. PW and FR contributed to the conception and design of the study as well as data analysis and writing of the manuscript.

## Conflict of Interest

The authors declare that the research was conducted in the absence of any commercial or financial relationships that could be construed as a potential conflict of interest.

## Publisher’s Note

All claims expressed in this article are solely those of the authors and do not necessarily represent those of their affiliated organizations, or those of the publisher, the editors and the reviewers. Any product that may be evaluated in this article, or claim that may be made by its manufacturer, is not guaranteed or endorsed by the publisher.

## Footnotes

1
http://neuromorpho.org/

2
https://www.python.org
