# Supplementary material for: Block Phenomena During Electric Micro-Stimulation of Pyramidal Cells and Retinal Ganglion Cells
Source: Front Cell Neurosci. 2021 Nov 26;15:771600. doi: 10.3389/fncel.2021.771600 (PMC8663762; doi:10.3389/fncel.2021.771600)
Supplement: Supplementary file 1 [file Data_Sheet_1.docx]

Supplementary Material

# Supplementary Figures

**Table S1:** r^2^ values for correlations between multiple geometric parameters and LT, UT as well as threshold ratio (UT/LT) in PCs. r^2^ values larger than 0.5 are indicated in yellow. AIS=Axon Initial Segment; Unmy=unmyelinated axon.

|  | 15 µm | | | 30 µm | | | 45 µm | | | 60 µm | | | 100 µm | | | 200 µm | | |
| --- | --- | --- | --- | --- | --- | --- | --- | --- | --- | --- | --- | --- | --- | --- | --- | --- | --- | --- |
|  | **LT** | **UT** | **UTLT** | **LT** | **UT** | **UTLT** | **LT** | **UT** | **UTLT** | **LT** | **UT** | **UTLT** | **LT** | **UT** | **UTLT** | **LT** | **UT** | **UTLT** |
| AIS  L | *0.21* | *0.34* | *0.16* | *0.33* | *0.05* | *0.01* | *0.29* | *0.00* | *0.29* | *0.30* | *0.00* | *0.37* | *0.30* | *0.03* | *0.18* | *0.38* | *0.12* | *0.04* |
| Unmy L | *0.01* | *0.04* | *0.04* | *0.01* | *0.04* | *0.02* | *0.05* | *0.08* | *0.02* | *0.07* | *0.00* | *0.00* | *0.11* | *0.00* | *0.01* | *0.06* | *0.04* | *0.04* |
| Axon L | *0.21* | *0.28* | *0.10* | *0.29* | *0.10* | *0.01* | *0.16* | *0.31* | *0.04* | *0.13* | *0.44* | *0.06* | *0.10* | *0.55* | *0.17* | *0.26* | *0.46* | *0.08* |
| Axon A | *0.04* | *0.64* | *0.44* | *0.01* | *0.46* | *0.14* | *0.01* | *0.22* | *0.02* | *0.01* | *0.08* | *0.01* | *0.02* | *0.06* | *0.04* | *0.00* | *0.01* | *0.00* |
| Dend L | *0.17* | *0.02* | *0.06* | *0.05* | *0.00* | *0.04* | *0.36* | *0.23* | *0.44* | *0.39* | *0.09* | *0.39* | *0.45* | *0.00* | *0.18* | *0.28* | *0.03* | *0.04* |
| Dend A | *0.42* | *0.03* | *0.00* | *0.47* | *0.01* | *0.14* | *0.79* | *0.00* | *0.61* | *0.78* | *0.03* | *0.33* | *0.82* | *0.33* | *0.03* | *0.82* | *0.39* | *0.01* |

**Table S2:** r^2^ values for correlations between multiple geometric parameters and LT, UT as well as threshold ratio (UT/LT) in RGCs. r^2^ values larger than 0.5 are indicated in yellow. Hill=hillock; AIS=Axon Initial Segment; DF=dendritic field.

|  | 15 µm | | | 30 µm | | | 45 µm | | | 60 µm | | | 100 µm | | | 200 µm | | |
| --- | --- | --- | --- | --- | --- | --- | --- | --- | --- | --- | --- | --- | --- | --- | --- | --- | --- | --- |
|  | **LT** | **UT** | **UTLT** | **LT** | **UT** | **UTLT** | **LT** | **UT** | **UTLT** | **LT** | **UT** | **UTLT** | **LT** | **UT** | **UTLT** | **LT** | **UT** | **UTLT** |
| Soma ⌀ | *0.02* | *0.67* | *0.43* | *0.13* | *0.09* | *0.00* | *0.03* | *0.00* | *0.01* | *0.01* | *0.01* | *0.00* | *0.01* | *0.01* | *0.02* | *0.03* | *0.00* | *0.07* |
| Hill  L | *0.06* | *0.09* | *0.03* | *0.23* | *0.00* | *0.05* | *0.20* | *0.04* | *0.15* | *0.18* | *0.01* | *0.05* | *0.23* | *0.05* | *0.10* | *0.36* | *0.02* | *0.21* |
| AIS  L | *0.28* | *0.26* | *0.37* | *0.19* | *0.38* | *0.39* | *0.28* | *0.12* | *0.25* | *0.28* | *0.03* | *0.24* | *0.27* | *0.01* | *0.21* | *0.15* | *0.08* | *0.13* |
| DF  ⌀ | *0.00* | *0.37* | *0.27* | *0.04* | *0.03* | *0.00* | *0.00* | *0.03* | *0.02* | *0.00* | *0.02* | *0.01* | *0.00* | *0.02* | *0.00* | *0.00* | *0.00* | *0.00* |
| Dend L | *0.02* | *0.20* | *0.18* | *0.00* | *0.15* | *0.11* | *0.04* | *0.00* | *0.01* | *0.07* | *0.03* | *0.00* | *0.09* | *0.05* | *0.02* | *0.05* | *0.00* | *0.03* |
| Dend A | *0.01* | *0.23* | *0.19* | *0.00* | *0.15* | *0.08* | *0.02* | *0.00* | *0.01* | *0.04* | *0.00* | *0.01* | *0.06* | *0.02* | *0.01* | *0.03* | *0.00* | *0.01* |
